# Epidemiology and associated factors of traumatic dental injuries presenting to emergency department: a retrospective study from Southern Taiwan

**DOI:** 10.1186/s12903-026-08746-0

**Published:** 2026-06-04

**Authors:** Po-Hsiang Chang, Sheng-Chun Chiu, Wei-Chih Chiu, Kai-Yun Tso

**Affiliations:** 1 Division of Endodontics, Department of Stomatology, College of Medicine, National Cheng Kung University Hospital, National Cheng Kung University, Tainan, 704302 Taiwan; 2 Department of Stomatology, College of Medicine, National Cheng Kung University Hospital, National Cheng Kung University, Tainan, 704302 Taiwan; 3 Institute of Oral Medicine, School of Dentistry, College of Medicine, National Cheng Kung University, Tainan, Taiwan

**Keywords:** Tooth injuries, Epidemiology, Accidents, Traffic, Emergency Service, Hospital

## Abstract

**Background:**

Traumatic dental injury (TDI) is a common dental emergency, yet prevalence data from Taiwan have relied mainly on questionnaire surveys. This study investigated TDI epidemiology in an emergency department setting and identified factors associated with combination injury, defined as the simultaneous presence of tooth hard tissue and periodontal injury.

**Methods:**

This retrospective cohort study included all patients with TDI affecting permanent dentition who presented to the Emergency Department of National Cheng Kung University Hospital, Tainan, Taiwan, between January and December 2014. Injuries were classified per the WHO 1978 and Andreasen 1981 systems. Associations were tested by chi-square with Monte Carlo simulation and multivariable binary logistic regression.

**Results:**

A total of 117 patients (72 males, 45 females; mean age 26.4 years) with 344 affected teeth were enrolled. Traffic accident was the leading cause (62.4%), predominantly motorcycle-related (63.0%). Upper central incisors were most frequently affected (45.3%). Crown fracture (23.0%) and subluxation (27.0%) were the most common injury types, and combination injury occurred in 18.0% of teeth. Multivariable logistic regression identified number of affected teeth (3–4 teeth: aOR 7.95, 95% CI 2.61–28.65; ≥5 teeth: aOR 11.99, 95% CI 2.97–56.57; both *p* < 0.001) and walk-in arrival (aOR 4.07, 95% CI 1.39–13.24, *p* = 0.013) as independent predictors, with good model discrimination (AUC 0.767, 95% CI 0.677–0.857).

**Conclusions:**

Emergency department TDI in Taiwan is dominated by motorcycle-related traffic accidents. Number of affected teeth and walk-in mode of arrival are independent predictors of combination injury. These findings support targeted motorcycle safety measures and comprehensive clinical and radiographic assessment for patients presenting with multiple affected teeth.

**Supplementary Information:**

The online version contains supplementary material available at 10.1186/s12903-026-08746-0.

## Background

Traumatic dental injury (TDI) has consistently been one of the most common dental emergencies. In 2016, Lam et al. defined TDI as an injury to the teeth and their associated soft and hard tissues resulting from impact to the oral cavity or its surrounding areas [1]. TDI typically occurs instantaneously and necessitates emergency treatment, followed by subsequent outpatient care and long-term follow-up.

The incidence and prevalence of TDI vary considerably across countries and age groups. According to a landmark meta-analysis incorporating 102 studies and 268,755 individuals, the global prevalence of TDI to permanent dentition is approximately 15.2% (95% CI: 13.0%–17.4%), with more than one billion living people estimated to have experienced a traumatic dental injury — a burden comparable to the world’s fifth most prevalent disease or injury [2]. These variations may be attributed to differences in the composition of study populations, research methodologies, or approaches to assessing variables. According to two previous Taiwanese studies on TDI prevalence [3, 4], the prevalence ranged from 16.5% to 19.9%, which exceeds the global average [5]. 

TDI represents one of the major categories of dental emergencies in hospital emergency departments. A systematic review and meta-analysis of 43 observational studies encompassing 209,099 patients found a pooled TDI prevalence of 15.4% (95% CI: 11%–21%) among all patients seeking emergency dental care globally, rising to 24% (95% CI: 15%–35%) in those under 21 years of age [6]. Despite this significant burden, emergency department-based TDI studies remain scarce in Asia, as in Taiwan. According to previous Taiwanese studies [7–10], pulp-related problems were the most common category of dental emergencies, while TDI accounted for approximately 20.9% to 22.2% of dental emergencies, ranking among the top conditions with a considerable proportion that should not be overlooked.

Previous Taiwanese TDI studies have relied exclusively on questionnaire-based surveys, which are susceptible to recall bias and limited to self-reported anterior dentition injury without clinical or radiographic confirmation [3, 4]. Consequently, the true clinical spectrum of TDI in the Taiwanese population remains incompletely characterized, and no emergency department-based epidemiological data currently exist. Globally, most TDI studies have been conducted in school-based settings or primary care clinics, with few examining emergency department presentations — particularly in Asian populations where motorcycle use constitutes a dominant mode of transportation. The present study was designed to address these gaps by using an emergency department setting with standardized clinical and radiographic examinations, and by incorporating the concept of combination injury — defined as the simultaneous presence of tooth hard tissue and periodontal injury — as an indicator of trauma severity [11–13].

This study had two primary objectives:

1. To investigate the epidemiological characteristics and patterns of traumatic dental injuries in an emergency department setting.
2. To evaluate trauma severity through combination injury and identify its associated risk factors.

We hypothesized that the occurrence of combination injury would be associated with specific etiological factors, patient demographics, and injury circumstances, which could inform targeted prevention strategies.

## Methods

### Study design and reporting guidelines

This retrospective cohort study was conducted and reported in accordance with the PROBE 2023 [14] (Preferred Reporting Items for Observational studies in Endodontics) guidelines. The completed PROBE checklist is provided as supplementary materials.

### Ethics and informed consent

Ethics approval was obtained from the Institutional Review Board of National Cheng Kung University Hospital (IRB No. A-ER-113-057); consent waiver and data handling details are provided in the Declarations.

### Sample size and patient selection

Based on pilot data indicating approximately 100 annual TDI cases at our institution, we used consecutive sampling to include all eligible cases during the one-year study period, ensuring representative data while minimizing selection bias. All patients presenting to the emergency department with TDI and meeting the inclusion criteria during the study period were consecutively enrolled.

### Inclusion and exclusion criteria

Inclusion criteria comprised patients who presented to the Emergency Department of National Cheng Kung University Hospital, Tainan, Taiwan, between January 1 and December 31, 2014, were clinically diagnosed with traumatic dental injury (TDI) resulting in varying degrees of permanent dentition damage, and had complete clinical examination, radiographic examination, and medical record data.

Exclusion criteria included: injuries to deciduous dentition; incomplete medical records or poor image quality preventing accurate interpretation; and cases with concomitant major systemic trauma affecting diagnostic assessment.

### Data sources and collection procedures

Data sources included the emergency medical information system and medical records of National Cheng Kung University Hospital. All clinical examinations were performed by licensed dentists who had received training in dental trauma management, including visual inspection, palpation, percussion testing, and electric pulp testing (EPT). Radiographic examinations included intraoral periapical radiographs and panoramic radiographs, with cone-beam computed tomography (CBCT) performed when necessary. Data collection utilized standardized forms to ensure consistency in data recording.

The following information was collected from each patient’s records: date of emergency visit, time of arrival at emergency department, mode of arrival at emergency department, age, gender, cause of accident, affected tooth position, number of affected teeth, initial electric pulp test results, initial pulpal diagnosis, type of fractured tooth, type of luxation injury, other diagnoses, and subsequent follow-up data including final electric pulp test results, final treatment modalities, and total number of follow-up visits. The definitions of each item mentioned above are described in the “Variables Definition” subsection below.

### Variables definition

Type of fractured tooth and luxation injury were classified per the WHO 1978 and Andreasen 1981 systems [5]. Fracture types comprised crown fracture, crown–root fracture, root fracture, and infraction; luxation types comprised concussion, subluxation, extrusive luxation, lateral luxation, intrusive luxation, and avulsion. Other diagnoses included mandibular, maxillary, and alveolar fractures and soft tissue laceration.

Combination injury was defined per Lauridsen et al. [11–13] as the simultaneous presence of both tooth hard tissue injury and periodontal injury, and was used — together with the number of affected teeth and final treatment modality — as an indicator of trauma severity.

Cause of accident was categorized as fall, traffic accident (sub-divided by vehicle type: car, motorcycle, bicycle, other), sports, violence, or other. Season followed the classification by Huang et al. [7]; time of arrival was grouped as morning, afternoon, evening, or night; mode of arrival as ambulance, walk-in, or other.

Affected tooth position used FDI notation. Electric pulp test (EPT) results were recorded as positive (sensation) or negative (no sensation), at both initial visit and final follow-up. Initial pulpal diagnosis was classified, based on radiographic findings of apical closure and root canal filling, as normal pulp, open apex, or previously treated. Final treatment was recorded as discontinue (DC), operative dentistry (OD), endodontic treatment (Endo), extraction, or surgery.

### Missing data handling

Descriptive statistical analysis was performed for missing data, recording the quantity and proportion of missing data for each variable. Statistical analysis employed the available case analysis method. For important variables such as electric pulp test results, if missing data exceeded 20%, sensitivity analysis was conducted to evaluate the impact on results.

### Statistical methods

All statistical analyses were performed using R version 4.5.3 (R Foundation for Statistical Computing, Vienna, Austria). Continuous variables were expressed as mean ± standard deviation (for normally distributed data) or median and interquartile range (for non-normally distributed data). Categorical variables were expressed as frequencies and percentages. Age was categorized into five groups (< 10, 11–20, 21–30, 31–40, and > 40 years) for descriptive analysis.

For between-group comparisons, categorical variables were analyzed using chi-square test with Monte Carlo simulation (50,000 simulations), which is appropriate when a substantial proportion of cells have expected counts below 5.

To identify independent predictors of combination injury, multivariable binary logistic regression was performed with combination injury (yes/no) as the dependent variable. Number of affected teeth (categorized as 1–2, 3–4, and ≥ 5), mode of arrival, age group (> 20 vs. ≤20 years), and cause of accident (traffic vs. non-traffic) were included as covariates. Model goodness-of-fit was assessed using the Hosmer-Lemeshow test, and discriminative ability was evaluated using the area under the receiver operating characteristic curve (AUC). Results are reported as adjusted odds ratios (OR) with 95% confidence intervals (CI). All statistical tests were two-tailed, with significance level set at α = 0.05. Of the 117 patients in the descriptive analyses, 15 were excluded from the regression model: 12 had alveolar fracture only without tooth-level injury (excluded a priori from all tooth-level analyses, as noted in the Table 1), and 3 had missing data on mode of arrival. The model was therefore performed on a complete-case sample of *n* = 102.

## Results

### Participants

From January to December 2014, a total of 117 patients with TDI presented to the emergency department of National Cheng Kung University Hospital. The cohort comprised 72 males and 45 females (male-to-female ratio: 1.6:1), with ages ranging from 6 to 77 years (mean age: 26.4 years).

### Cause of accident

Traffic accident was the predominant cause (62.4%), followed by sports (17.1%), fall down (12.0%), and violence (2.6%). Within traffic accident, motorcycles were by far the most common vehicle type (63.0%), followed by bicycles (15.1%) and cars (6.8%). The distribution of all variables by cause of accident is presented in Table 1.

**Table 1:** Distribution of variables by cause of accident

Variable	Fall	Traffic	Sports	Violence	Others	Total	*p*-valueᵃ
Patient-level variables (*n* = 117)							
Gender							< 0.001**
Male	8 (6.8%)	36 (30.8%)	20 (17.1%)	2 (1.7%)	6 (5.1%)	72 (61.5%)	
Female	6 (5.1%)	37 (31.6%)	0	1 (0.9%)	1 (0.9%)	45 (38.5%)	
Total	14 (12.0%)	73 (62.4%)	20 (17.1%)	3 (2.6%)	7 (5.9%)	117	
Age							0.012*
<10	3 (2.6%)	3 (2.6%)	0	0	3 (2.6%)	9 (7.7%)	
11–20	3 (2.6%)	30 (25.6%)	12 (10.3%)	0	2 (1.7%)	47 (40.2%)	
21–30	2 (1.7%)	19 (16.2%)	8 (6.8%)	1 (0.9%)	0	30 (25.6%)	
31–40	1 (0.9%)	11 (9.4%)	0	0	1 (0.9%)	13 (11.1%)	
>41	5 (4.3%)	10 (8.5%)	0	2 (1.7%)	1 (0.9%)	18 (15.4%)	
Total	14 (12.0%)	73 (62.4%)	20 (17.1%)	3 (2.6%)	7 (5.9%)	117	
Season							0.692 NS
Spring	4 (3.4%)	14 (12.0%)	7 (6.0%)	0	1 (0.9%)	26 (22.2%)	
Summer	2 (1.7%)	11 (9.4%)	5 (4.3%)	1 (0.9%)	1 (0.9%)	20 (17.1%)	
Autumn	5 (4.3%)	24 (20.5%)	5 (4.3%)	0	3 (2.6%)	37 (31.6%)	
Winter	3 (2.6%)	24 (20.5%)	3 (2.6%)	2 (1.7%)	2 (1.7%)	34 (29.1%)	
Total	14 (12.0%)	73 (62.4%)	20 (17.1%)	3 (2.6%)	7 (5.9%)	117	
Time of arrival							0.033*
Morning	4 (3.4%)	21 (18.0%)	3 (2.6%)	2 (1.7%)	1 (0.9%)	31 (26.5%)	
Afternoon	3 (2.6%)	17 (14.5%)	10 (8.5%)	0	0	30 (25.6%)	
Evening	3 (2.6%)	26 (22.2%)	7 (6.0%)	0	4 (3.4%)	40 (34.2%)	
Night	3 (2.6%)	4 (3.4%)	0	1 (0.9%)	0	8 (6.8%)	
No record	1 (0.9%)	5 (4.3%)	0	0	2 (1.7%)	8 (6.8%)	
Total	14 (12.0%)	73 (62.4%)	20 (17.1%)	3 (2.6%)	7 (5.9%)	117	
Mode of arrival							0.006**
Ambulance	6 (5.1%)	52 (44.4%)	8 (6.8%)	2 (1.7%)	2 (1.7%)	70 (59.8%)	
Walk-in	6 (5.1%)	12 (10.3%)	11 (9.4%)	1 (0.9%)	2 (1.7%)	32 (27.4%)	
No record	2 (1.7%)	9 (7.7%)	1 (0.9%)	0	3 (2.6%)	15 (12.8%)	
Total	14 (12.0%)	73 (62.4%)	20 (17.1%)	3 (2.6%)	7 (5.9%)	117	
Number of affected teeth							0.071 NS
0	1 (0.9%)	10 (8.5%)	1 (0.9%)	0	0	12 (10.3%)	
1	1 (0.9%)	3 (2.6%)	5 (4.3%)	0	3 (2.6%)	12 (10.3%)	
2	3 (2.6%)	15 (12.8%)	7 (6.0%)	2 (1.7%)	2 (1.7%)	29 (24.8%)	
3	4 (3.4%)	20 (17.1%)	4 (3.4%)	0	2 (1.7%)	30 (25.6%)	
4	1 (0.9%)	13 (11.1%)	2 (1.7%)	0	0	16 (13.7%)	
≥5	4 (3.4%)	12 (10.3%)	1 (0.9%)	1 (0.9%)	0	18 (15.4%)	
Total	14 (12.0%)	73 (62.4%)	20 (17.1%)	3 (2.6%)	7 (5.9%)	117	
Number of follow-up visits							0.218 NS
0	1 (0.9%)	4 (3.4%)	3 (2.6%)	1 (0.9%)	0	9 (7.7%)	
1	2 (1.7%)	16 (13.7%)	1 (0.9%)	0	0	19 (16.2%)	
2–4	4 (3.4%)	33 (28.2%)	10 (8.5%)	2 (1.7%)	3 (2.6%)	52 (44.4%)	
>5	7 (6.0%)	20 (17.1%)	6 (5.1%)	0	4 (3.4%)	37 (31.6%)	
Total	14 (12.0%)	73 (62.4%)	20 (17.1%)	3 (2.6%)	7 (5.9%)	117	
Tooth-level variables (*n* = 344)							
Affected tooth position							0.040*
Upper central incisor	20 (5.8%)	101 (29.4%)	24 (7.0%)	1 (0.3%)	10 (2.9%)	156 (45.3%)	
Upper lateral incisor	13 (3.8%)	62 (18.0%)	9 (2.6%)	2 (0.6%)	2 (0.6%)	88 (25.6%)	
Upper canine	4 (1.2%)	14 (4.1%)	0	1 (0.3%)	0	19 (5.5%)	
Lower central incisor	4 (1.2%)	23 (6.7%)	6 (1.7%)	3 (0.9%)	1 (0.3%)	37 (10.8%)	
Lower lateral incisor	2 (0.6%)	18 (5.2%)	5 (1.5%)	4 (1.2%)	0	29 (8.4%)	
Lower canine	0	4 (1.2%)	0	1 (0.3%)	0	5 (1.5%)	
Others	4 (1.2%)	5 (1.5%)	1 (0.3%)	0	0	10 (2.9%)	
Total	47 (13.7%)	227 (66.0%)	45 (13.1%)	12 (3.5%)	13 (3.8%)	344	
Type of fractured tooth							0.006**
Crown fracture	17 (4.9%)	55 (16.0%)	7 (2.0%)	1 (0.3%)	2 (0.6%)	82 (23.8%)	
Root fracture	2 (0.6%)	8 (2.3%)	4 (1.2%)	1 (0.3%)	2 (0.6%)	17 (4.9%)	
Crown-root fracture	4 (1.2%)	4 (1.2%)	2 (0.6%)	3 (0.9%)	0	13 (3.8%)	
No record	24 (7.0%)	160 (46.5%)	32 (9.3%)	7 (2.0%)	9 (2.6%)	232 (67.4%)	
Total	47 (13.7%)	227 (66.0%)	45 (13.1%)	12 (3.5%)	13 (3.7%)	344	
Type of luxation injury							0.055 NS
Concussion	5 (1.5%)	30 (8.7%)	6 (1.7%)	0	3 (0.9%)	44 (12.8%)	
Subluxation	11 (3.2%)	62 (18.0%)	12 (3.5%)	7 (2.0%)	4 (1.2%)	96 (27.9%)	
Extrusive luxation	2 (0.6%)	20 (5.8%)	9 (2.6%)	1 (0.3%)	1 (0.3%)	33 (9.6%)	
Lateral luxation	8 (2.3%)	39 (11.3%)	10 (2.9%)	0	3 (0.9%)	60 (17.4%)	
Intrusive luxation	5 (1.5%)	9 (2.6%)	2 (0.6%)	0	0	16 (4.7%)	
Avulsion	2 (0.6%)	31 (9.0%)	1 (0.3%)	0	1 (0.3%)	35 (10.2%)	
No record	14 (4.1%)	36 (10.5%)	5 (1.5%)	4 (1.2%)	1 (0.3%)	60 (17.4%)	
Total	47 (13.7%)	227 (66.0%)	45 (13.1%)	12 (3.5%)	13 (3.7%)	344	
Initial pulpal diagnosis							< 0.001**
Normal pulp	36 (10.5%)	190 (55.2%)	41 (11.9%)	12 (3.5%)	3 (0.9%)	282 (82.0%)	
Open apex	6 (1.7%)	14 (4.1%)	0	0	9 (2.6%)	29 (8.4%)	
Previously treated	5 (1.5%)	8 (2.3%)	1 (0.3%)	0	1 (0.3%)	15 (4.4%)	
No record	0	15 (4.4%)	3 (0.9%)	0	0	18 (5.2%)	
Total	47 (13.7%)	227 (66.0%)	45 (13.1%)	12 (3.5%)	13 (3.7%)	344	
Initial EPT							0.033*
EPT (+)	7 (2.0%)	98 (28.5%)	15 (4.4%)	0	3 (0.9%)	123 (35.8%)	
EPT (−)	10 (2.9%)	68 (19.8%)	20 (5.8%)	4 (1.2%)	1 (0.3%)	103 (29.9%)	
No record	30 (8.7%)	61 (17.7%)	10 (2.9%)	8 (2.3%)	9 (2.6%)	118 (34.3%)	
Total	47 (13.7%)	227 (66.0%)	45 (13.1%)	12 (3.5%)	13 (3.7%)	344	
Final EPT							0.007**
EPT (+)	5 (1.5%)	88 (25.6%)	16 (4.7%)	2 (0.6%)	3 (0.9%)	114 (33.1%)	
EPT (−)	18 (5.2%)	59 (17.2%)	20 (5.8%)	2 (0.6%)	2 (0.6%)	101 (29.4%)	
No record	24 (7.0%)	80 (23.3%)	9 (2.6%)	8 (2.3%)	8 (2.3%)	129 (37.5%)	
Total	47 (13.7%)	227 (66.0%)	45 (13.1%)	12 (3.5%)	13 (3.7%)	344	

The high proportion of unrecorded luxation type (17.4%) likely reflects the inherent diagnostic difficulty of luxation injuries in the emergency department setting.

Twelve patients presented with alveolar fracture only, without direct tooth hard tissue or periodontal injury, and were recorded as having zero affected teeth. These patients were included in Table 1 but were excluded from tooth-level analyses (Tables 3 and 4).

*EPT* Electric pulp test, *NS*  Not significant, * *p* < 0.05; ** *p* < 0.01

ᵃp-values were calculated using chi-square test with Monte Carlo simulation (50,000 simulations). For variables with missing records (Time of arrival, Mode of arrival, Type of fractured tooth, Type of luxation injury, Initial pulpal diagnosis, Initial EPT, Final EPT), p-values were calculated among cases with available records only.

### Gender and age

Both gender and age were significantly associated with cause of accident (*p* < 0.001 and *p* = 0.012, respectively; Table 1). Sports injuries occurred exclusively in male patients and were concentrated in the 11–20 age group, whereas traffic accidents showed a balanced sex distribution.

### Season and time of arrival

Autumn had the highest seasonal incidence (31.6%), but seasonal distribution was not significantly associated with cause of accident (*p* = 0.695; Table 1). Time of arrival was significantly associated with cause (*p* = 0.033; Table 1), with sports injuries concentrated in the afternoon; this result should be interpreted with caution given the low expected counts in several cells.

### Mode of arrival

Mode of arrival was significantly associated with cause of accident (*p* = 0.006; Table 1): ambulance arrivals were predominantly traffic accident cases, whereas walk-in patients more frequently presented with sports injuries.

### Number of affected teeth

The 117 patients had 344 affected teeth (mean 2.94 per patient); an additional 12 patients (10.3%) presented with alveolar fracture only and no tooth-level injury (Table 1 footnote). Number of affected teeth was not significantly associated with cause of accident (*p* = 0.071; Table 1).

### Number of follow-up visits

Number of follow-up visits was not significantly associated with cause of accident (*p* = 0.215; Table 1).

### Type of fractured tooth

Crown fracture was the most common fracture type (Table 1). Fracture type distribution differed significantly by cause of accident (*p* = 0.006), with crown–root fractures over-represented in violence-related injuries and root fractures in sports injuries.

### Type of luxation injury

Subluxation was the most common luxation type (Table 1). Luxation type could not be recorded in 17.4% of teeth, likely reflecting the inherent diagnostic difficulty of luxation assessment in the emergency setting. The distribution across causes of accident did not reach statistical significance (*p* = 0.055).

### Initial pulpal diagnosis

Initial pulpal diagnosis differed significantly by cause of accident (*p* < 0.001; Table 1). Open apex cases were disproportionately represented in the “Others” category, whereas sports injuries contained no open apex cases — consistent with the older age distribution of sports-related TDI.

### Electric pulp test (EPT)

Initial EPT results showed a marginally significant association with cause of accident (*p* = 0.033; Table 1); however, EPT missing rates differed markedly by injury type (fall-down 63.8% vs. traffic 26.9%), suggesting differential clinical feasibility and warranting cautious interpretation. Final EPT results were significantly associated with cause (*p* = 0.007; Table 1), with traffic accident showing the highest proportion of EPT-negative outcomes, suggesting greater risk of post-traumatic pulp necrosis.

### Tooth position distribution

Upper central incisors were the most frequently affected position (45.3%; Table 2). Upper jaw teeth collectively accounted for 76.5% of affected positions, significantly higher than the expected 50% under equal jaw distribution (*p* < 0.001).

**Table 2:** Distribution of affected tooth positions

Tooth Position	*n* (Teeth)	Percent (%)	*p*-value
Upper jaw	263	76.5	< 0.001
Upper central incisor	156	45.3	
Upper lateral incisor	88	25.6	
Upper canine	19	5.5	
Lower jaw / Others	81	23.5	
Lower central incisor	37	10.8	
Lower lateral incisor	29	8.4	
Lower canine	5	1.5	
Others	10	2.9	
Total	344	100.0	

Percentages are based on 344 teeth with available position records (12 teeth with no recorded position excluded)

FDI notation: upper central incisor = teeth 11, 21; upper lateral incisor = teeth 12, 22; upper canine = teeth 13, 23; lower central incisor = teeth 31, 41; lower lateral incisor = teeth 32, 42; lower canine = teeth 33, 43; others = teeth 14, 15, 24, 26, 34, 44

* p-value from one-sample proportion test comparing upper jaw (76.5%) vs. lower jaw/others (23.5%); *p* < 0.001

### Combination injury

**Fig. 1:**
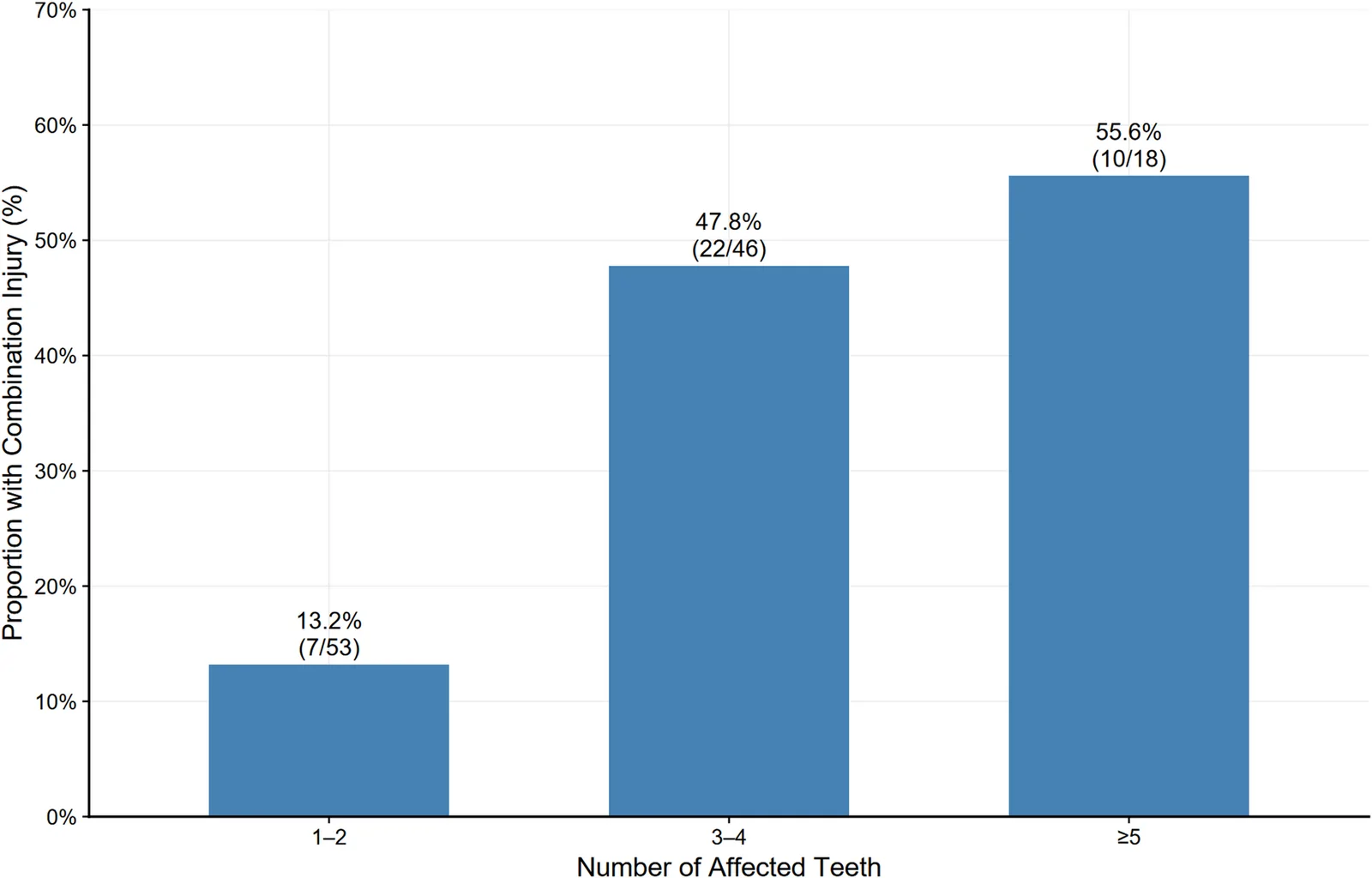
Combination injury rate by number of affected teeth

Combination injury was present in 62 teeth (18.0%), distributed among 39 patients (33.3%). Univariate analysis identified number of affected teeth (*p* < 0.001) and mode of arrival (*p* = 0.036) as the only variables significantly associated with combination injury (Table 3); a clear dose-response pattern was observed for number of affected teeth (Fig. 1). Other variables — including gender, age, season, cause of accident, time of arrival, EPT results, and follow-up visits — were not significantly associated (Table 3).

**Table 3:** Univariate analysis of factors associated with combination injury

	Combination injury		
	Yes	No	*p-value*
Gender			0.687
Male Female	25/72 (34.7%) 14/45 (31.1%)	47/72 (65.3%) 31/45 (68.9%)	
Age			0.532
< 10 11–20 21–30 31–40 > 41	4/9 (44.4%) 14/47 (29.8%) 13/30 (43.3%) 4/13 (30.8%) 4/18 (22.2%)	5/9 (55.6%) 33/47 (70.2%) 17/30 (56.7%) 9/13 (69.2%) 14/18 (77.8%)	
Season			0.389
Spring (2–4) Summer (5–7) Fall (8–10) Winter (11 − 1)	10/26 (38.5%) 9/20 (45.0%) 12/37 (32.4%) 8/34 (23.5%)	16/26 (61.5%) 11/20 (55.0%) 25/37 (67.6%) 26/34 (76.5%)	
Cause of accident			0.653
Fall Traffic Sports Violence Others	7/14 (50.0%) 24/73 (32.9%) 5/20 (25.0%) 1/3 (33.3%) 2/7 (28.6%)	7/14 (50.0%) 49/73 (67.1%) 15/20 (75.0%) 2/3 (66.7%) 5/7 (71.4%)	
Time of arrival			0.833
Morning Afternoon Evening Night No record	10/31 (32.3%) 9/30 (30.0%) 14/40 (35.0%) 4/8 (50.0%) 2/8 (25.0%)	21/31 (67.7%) 21/30 (70.0%) 26/40 (65.0%) 4/8 (50.0%) 6/8 (75.0%)	
Mode of arrival			0.036
Ambulance Walk in No record	20/70 (28.6%) 16/32 (50.0%) 3/15 (20.0%)	50/70 (71.4%) 16/32 (50.0%) 12/15 (80.0%)	
Number of affected teeth (*n* = 105)			< 0.001
1 2 3 4 ≧ 5	1/12 (8.3%) 6/29 (20.7%) 14/30 (46.7%) 8/16 (50.0%) 10/18 (55.6%)	11/12 (91.7%) 23/29 (79.3%) 16/30 (53.3%) 8/16 (50.0%) 8/18 (44.4%)	
Initial EPT			0.171
EPT (+) EPT (-) No record	23/123 (18.7%) 12/103 (11.7%) 27/118 (22.9%)	100/123 (81.3%) 91/103 (88.3%) 91/118 (77.1%)	
Final EPT			0.918
EPT (+) EPT (-) No record	19/114 (16.7%) 17/101 (16.8%) 26/129 (20.2%)	95/114 (83.3%) 84/101 (83.2%) 103/129 (79.8%)	
Number of follow-up visits			0.829
0 1 2–4 > 5	3/9 (33.3%) 8/19 (42.1%) 17/52 (32.7%) 11/37 (29.7%)	6/9 (66.7%) 11/19 (57.9%) 35/52 (67.3%) 26/37 (70.3%)	

p-values were calculated using chi-square test with Monte Carlo simulation (50,000 simulations) among cases with available records only

### Multivariate analysis of combination injury

**Fig. 2:**
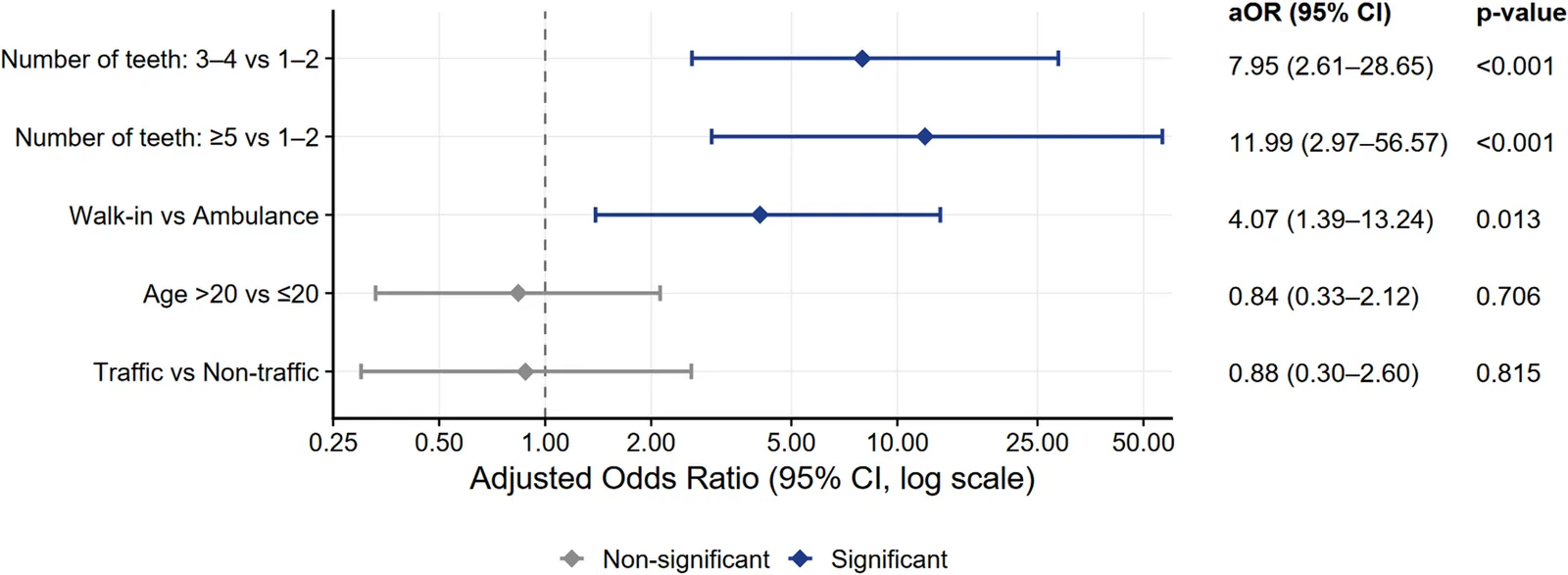
Forest plot: independent predictors of combination injuries

**Fig. 3:**
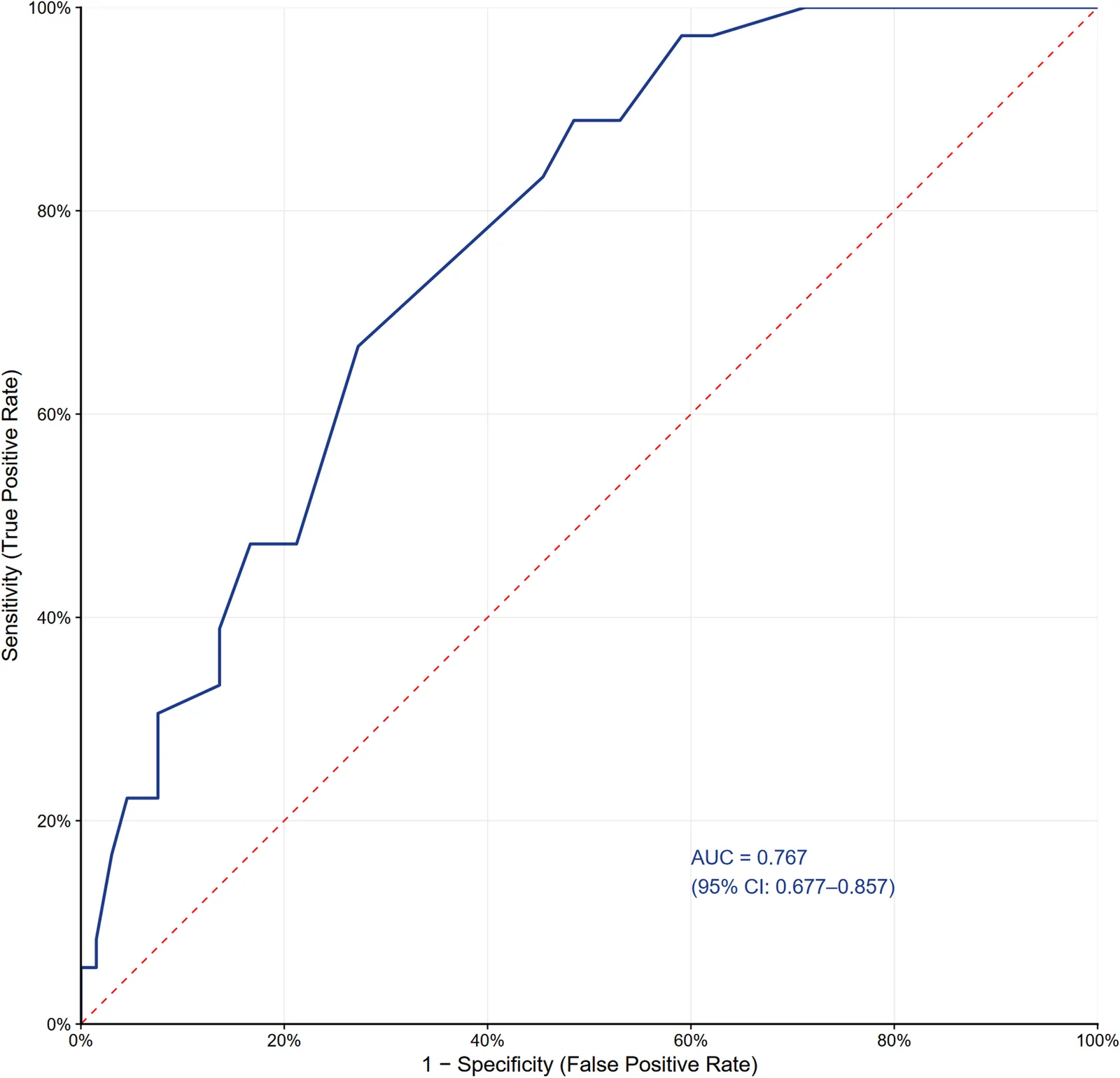
ROC curve: prediction of combination injury

Multivariable logistic regression (*n* = 102) confirmed that number of affected teeth and walk-in mode of arrival remained independent predictors of combination injury after adjustment, with a clear dose-response across the number-of-affected-teeth categories (Table 4; Fig. 2). Age group and cause of accident were not independently associated with combination injury. The model showed good fit (Hosmer-Lemeshow *p* = 0.611) and good discriminative ability (AUC = 0.767, 95% CI: 0.677–0.857; Fig. 3).

**Table 4:** Multivariable logistic regression for combination injury predictors (*n* = 102)

Variable	Adjusted OR	95% CI	*p*-value
Teeth 3–4 vs. 1–2	7.95	2.61–28.65	< 0.001*
Teeth ≥ 5 vs. 1–2	11.99	2.97–56.57	< 0.001*
Walk-in vs. Ambulance	4.07	1.39–13.24	0.013*
Age > 20 vs. ≤ 20	0.84	0.33–2.12	0.718
Traffic vs. Non-traffic	0.88	0.30–2.60	0.820

Adjusted for number of affected teeth, mode of arrival, age group, and cause of accident. Reference groups: 1–2 teeth, ambulance, ≤20 years, non-traffic. *OR* Odds ratio, *CI* Confidence interval

### Final treatment

Final treatment modalities included: 16 teeth (4.7%) received operative dentistry (OD), 91 teeth (26.5%) received endodontic treatment (Endo), 12 teeth (3.5%) underwent extraction, 15 teeth or related sites (4.4%) underwent surgery, and 219 teeth were classified as discontinue (DC).

### Missing data and sensitivity analysis

The proportion of missing data for main variables in this study was as follows: initial electric pulp test (EPT) 118/344 (34.3%), final EPT 129/344 (37.5%). The primary reasons for missing data were patients not returning for follow-up (*n* = 85, 72.0%), teeth extracted or surgically treated making testing impossible (*n* = 21, 17.8%), and poor patient cooperation (*n* = 12, 10.2%).

Sensitivity analysis comparing baseline characteristics between the group with complete EPT records (*n* = 215) and the missing data group (*n* = 129) showed no statistically significant differences in age (26.8 ± 14.2 vs. 25.9 ± 13.7 years, *p* = 0.612), gender ratio (*p* = 0.534), distribution of causes of injury (*p* = 0.428), or mean number of affected teeth (2.9 ± 1.8 vs. 3.1 ± 2.0 teeth, *p* = 0.371), indicating that the missing data were random and had limited impact on the main study results.

## Discussion

This retrospective epidemiological study was based on a comprehensive analysis of clinical records of TDI patients treated in the dental emergency department over a one-year period. Since National Cheng Kung University Hospital is one of only two hospitals in Tainan City that accepts dental emergencies, patients with dental emergency needs in this region, regardless of ethnicity, are highly likely to seek treatment at our institution, reducing bias toward a specific population. Compared to two previous Taiwanese studies [3, 4] that adopted questionnaire surveys combined with clinical examination, where the events may have occurred in the more distant past, our study recorded the current status in the emergency department, providing more immediate data. Additionally, previous studies were conducted in classroom settings and only examined the anterior teeth, whereas our study involved clinical examination combined with radiographic examination, potentially offering better accuracy. The exclusion of deciduous dentition and cases with concomitant major systemic trauma was implemented to reduce confounding variables.

The male predominance observed in our cohort is consistent with the epidemiological range of 1.3–2.3:1 reported globally [15], and has been attributed to higher participation in sports and violence-related activities among males. Within our cohort, however, gender distribution differed markedly by injury subtype: traffic accidents showed no significant sex difference, whereas sports injuries were highly male-predominant (*p* < 0.001), supporting a gender-sport interaction consistent with prior reports.

TDI classically peaks in the 6–23 year range, attributed to developing mobility and incompletely matured motor coordination [15, 16]. Our cohort showed a similar peak in the 11–20 age group, which also corresponded to the age range most affected by traffic accidents — the dominant injury cause in this study — likely contributing to the elevated mean age.

The predominance of traffic accidents in our cohort contrasts with most international studies [17, 18], in which falls or sports injuries are the leading cause. This discrepancy likely reflects unique features of Taiwan’s transportation landscape: according to official statistics from the Directorate General of Highways, motorcycles have consistently accounted for approximately 63–65% of all registered vehicles in Taiwan throughout the study period and beyond [19]. A similar motorcycle-dominant pattern of TDI etiology has been reported across Southeast Asian countries [20]. The combination of high motorcycle use and rider vulnerability likely explains the elevated proportion of traffic-related TDI in this cohort, and underscores the need for region-specific prevention strategies targeting motorcycle safety — particularly for the 11–20 age group, which demonstrated the highest TDI incidence in the present study.

In contrast to most published studies that report higher TDI incidence in warmer months [21–23], our data showed higher incidence in autumn (31.6%) and winter (29.1%). This may reflect the predominance of traffic accidents in our cohort, as reduced visibility and road conditions in cooler months may contribute to increased motorcycle-related incidents. However, seasonal distribution was not significantly associated with cause of accident (Monte Carlo *p* = 0.695), and this pattern should be interpreted cautiously given the single-year data collection window.

The evening peak in TDI presentations aligns with peak commuting traffic density; a similar evening–night clustering has been reported for two-wheeler-related dental trauma under conditions of reduced visibility [24]. Motorcycle safety and road illumination improvements during evening hours may therefore be particularly relevant for motorcycle-dominant environments such as southern Taiwan.

The predominance of maxillary anterior teeth, with upper central and lateral incisors most commonly affected (Table 2), is consistent with prior reports [17, 25, 26]. Their anatomical prominence — often accompanied by increased overjet and inadequate lip coverage — has been established as a biomechanical risk factor for TDI [18].

In the TDI cases evaluated in this study, only 10.3% of patients had only one tooth involved, while 79.4% had two or more teeth affected, with the maximum being nine teeth. This is consistent with findings from previous surveys [26], confirming that most injuries involve multiple teeth. Other studies have indicated that the number of affected teeth appears to alter the severity of TDI depending on the cause of trauma; for example, motorcycle-related TDI in traffic accidents is often more severe compared to bicycle-related TDI and may increase the number of affected teeth [27]. Our study shows that the incidence of traffic accident-related TDI is relatively high in Taiwan, reminding us that relevant authorities should emphasize preventive measures for traffic accidents.

Crown fracture and subluxation were the most common fracture and luxation injury types, respectively, consistent with prior reports and a systematic review by Azami-Aghdash et al. [26, 28, 29]. The relatively low proportion of concussion compared with other studies [5] may reflect that patients with milder symptoms often do not present to the emergency department and are instead managed in primary care settings.

Lauridsen et al. [11–13] characterized combination injury as a more severe form of trauma with higher risk of complex healing and subsequent pulp necrosis. The combination injury prevalence observed in our cohort may underestimate the true rate, as luxation type could not be confirmed in 17.4% of affected teeth owing to the diagnostic constraints of the emergency department setting; such cases were excluded from combination injury classification.

The association between combination injury and number of affected teeth is further corroborated by a 20-year retrospective study from a specialized dental trauma center in Brazil (*n* = 837 patients, 2,357 teeth), which found that patients with three or more affected teeth were significantly more likely to suffer moderate or severe TDI compared to those with one or two affected teeth (adjusted OR for severe injury = 4.64, 95% CI: 3.11–6.93, *p* < 0.001 [30]. The biomechanical explanation is consistent: a higher number of affected teeth implies a greater magnitude of traumatic force applied, which in turn increases the probability of simultaneous hard tissue and periodontal injury — the defining characteristic of combination injury. Clinicians should therefore maintain high awareness for combination injuries in patients with multiple affected teeth, as these cases require more intensive follow-up and monitoring for pulp necrosis. The multivariable logistic regression confirmed this independent, dose-dependent association after adjustment for mode of arrival, age, and cause of accident (Table 4).

The independent association between walk-in arrival and combination injury (Table 4) should be considered hypothesis-generating rather than definitive. Two non-mutually-exclusive mechanisms may contribute. First, sports-related injuries were disproportionately common among walk-in patients (Table 1); although often not requiring emergency transport, such injuries can involve sufficient localized impact to produce combination injuries. Second, ambulance-transported patients with severe multi-system trauma may receive less thorough dental examination because clinical attention is directed toward life-threatening injuries — a pattern supported by recent polytrauma literature: Lucarelli et al. (2025) reported a 76% missed-diagnosis rate for dental trauma in high-energy polytrauma patients [31], and Meyer et al. (2022) found that only 11.8% of dental injuries on whole-body trauma CT were captured in original radiology reports [32]. These findings support the plausibility of underdiagnosis in our ambulance-arrived group, but prospective studies with standardized dental examination across all triage categories are needed to confirm this mechanism.

This study has several limitations. First, data were collected over a single year (2014); a 12-month window may be susceptible to year-specific factors that could influence apparent seasonal and time-of-arrival distributions. The total number of TDI patients screened was not recorded, limiting formal assessment of selection bias. Second, the regression was performed on a complete-case sample (*n* = 102), excluding 12 patients with alveolar fracture only and 3 with missing mode of arrival. Complete-case analysis can introduce bias if missingness is informative; however, baseline characteristics did not differ systematically between excluded and retained patients, and the absolute exclusion rate (3 of 105, 2.9%) is unlikely to materially distort the estimates. Third, EPT data had > 30% missing values due to loss to follow-up, extraction or surgical removal, and inability to cooperate in the ED setting. Multiple imputation was not applied because missingness was unlikely to be at random — each cause represents a clinically distinct subgroup rather than a random subset. As EPT is a secondary descriptive variable not used in the regression model, the primary conclusions are not materially affected; however, EPT-based pulpal outcomes should be interpreted with caution. Fourth, extraction was incompletely documented and could not be evaluated as a severity indicator. Finally, as a single-center ED cohort, the study over-represents severe cases and under-represents milder TDI managed in community settings; the motorcycle-dominant cause-of-accident pattern is specific to southern Taiwan and may not generalize to other regions. Multi-center prospective studies with larger samples, longer follow-up, broader geographic coverage, and structured EPT protocols applied at standardized follow-up intervals are needed to validate these findings and develop region-specific prevention strategies.

## Conclusion

In this emergency department–based cohort from southern Taiwan, TDI was dominated by motorcycle-related traffic accidents, with peak incidence among males and adolescents aged 11–20 years; maxillary anterior teeth were disproportionately affected. Multivariable logistic regression confirmed that number of affected teeth and walk-in mode of arrival are independent predictors of combination injury. These findings support region-specific prevention strategies — particularly motorcycle safety interventions targeting the 11–20 age group — and comprehensive clinical and radiographic assessment for patients presenting with multiple affected teeth.

## Supplementary information

Supplementary Material 1.

## Data Availability

The data that support the findings of this study are available from the corresponding author upon reasonable request. The data are not publicly available due to privacy and ethical restrictions.

## Abbreviations

TDI: Traumatic dental injury
EPT: Electric pulp test
DC: Discontinue
OD: Operative dentistry
Endo: Endodontic treatment
WHO: World Health Organization
PROBE: Preferred Reporting Items for Observational studies in Endodontics
IRB: Institutional Review Board
CBCT: Cone-beam computed tomography
FDI: FDI World Dental Federation
